# Unveiling the structure and interactions of silicene grown on MoS_2_: insights from hybrid molecular dynamics simulations

**DOI:** 10.1107/S2052520625009187

**Published:** 2025-11-10

**Authors:** Alexandre Melhorance Barboza, Luis César Rodríguez Aliaga, Daiara Fernandes de Faria, Ivan Napoleão Bastos

**Affiliations:** a Department of Computational Modeling Rio de Janeiro State University (UERJ) Polytechnic Institute (IPRJ) 25 Bonfim Street Vila Amélia Nova Friburgo RJ 28625-570 Brazil; Georgetown University, USA

**Keywords:** epitaxial growth, molecular dynamics, silicene, molybdenum disulfide, van der Waals interactions

## Abstract

The interaction of silicene with MoS_2_ surfaces has an important role in determining many properties of these systems. However, the intricate nature of silicene-based systems presents significant challenges for both experimental and computational investigations, often resulting in conflicting reports in the literature. Using molecular dynamics simulations with hybrid potentials, this work investigates the epitaxial growth of silicene on an MoS_2_ substrate using a vapor-deposition-like method. The findings reveal that silicene can achieve stability on an MoS_2_ substrate in an *AB* low-buckled configuration when both van der Waals and covalent interactions are considered. In this configuration, the silicene layer exhibits numerous defects, which can be significantly reduced by increasing the substrate temperature. In contrast, when only van der Waals interactions are accounted for, the system becomes unstable, leading to the collapse of the silicene structure into a three-dimensional disordered arrangement of Si atoms. A similar instability is observed in a heterostructure where a silicene sheet is intercalated between two MoS_2_ layers. These results, combined with the lattice mismatch observed between silicene and MoS_2_, suggest that van der Waals interactions alone are insufficient to ensure the mechanical stability of silicene.

## Abstract 2

The stable epitaxial growth of silicene on MoS_2_ requires both van der Waals and covalent interactions – without them, the silicene collapses into a disordered state. These findings offer important insights into designing stable silicene/MoS_2_ heterostructures for future nanotechnology applications.

## 1. Introduction

As society becomes increasingly dependent on advanced technology for communication, energy, healthcare and transportation, the demand for improved materials and innovations continues to grow. Existing materials are reaching their physical limits, hindering further progress. For instance, current transistor technology, primarily based on silicon, faces significant limitations as device dimensions approach the nanoscale. As transistors shrink, issues such as quantum tunneling, short-channel effects and heat dissipation become more prominent, severely impacting performance and energy efficiency (Franklin, 2015 ▸). The traditional scaling method, as described by Moore’s Law (Theis & Wong, 2017 ▸), is reaching its physical and economic limits, making it increasingly difficult to improve transistor speed and power consumption. Therefore, to meet the ever-growing demand for faster, smaller and more efficient devices, as well as more sustainable and durable solutions, new material technologies must be developed.

In the last decade, nanotechnology has emerged as one of the promising solutions to address these challenges, offering engineered nanomaterials with great potential for producing products with substantially improved performance (Baig *et al.*, 2021 ▸). The field of nanotechnology is vast and encompasses a broad range of applications, from agriculture to pharmaceuticals (Ajaz *et al.*, 2024 ▸). In the specific application within the semiconductor field, nanomaterials, especially two-dimensional (2D) ones, are of great interest for use as transistors due to their enhanced properties compared with their conventional materials counterparts (Franklin, 2015 ▸).

The precursor to 2D materials, graphene, lacks a practical bandgap for transistor switching, motivating the search for other 2D materials, such as silicene, which is compatible with current silicon-based semiconductor technology and has already shown potential as a transistor (Emami-Nejad *et al.*, 2023 ▸; Tao *et al.*, 2015 ▸). However, the high air reactivity of silicene, caused by its buckled structure, makes device fabrication extremely difficult, as freestanding silicene quickly collapses (Peplow, 2015 ▸); therefore, it is typically synthesized via epitaxial growth on a substrate, which provides the necessary stability to prevent degradation (Kharadi *et al.*, 2020 ▸).

To date, silicene has been grown on various substrates, primarily noble metals (Vogt *et al.*, 2012 ▸; Meng *et al.*, 2013 ▸; Stepniak-Dybala & Krawiec, 2019 ▸). However, characterizing the electronic and electrical properties of silicene on metallic substrates is highly challenging, as these properties are largely dominated by the metal. Additionally, the interaction with a metallic substrate may significantly depress the electronic properties of silicene, making potential applications in nanoelectronic devices unviable (Houssa *et al.*, 2015 ▸). Therefore, finding ways to eliminate or minimize substrate effects has become a critical challenge in the development of silicene-based devices (Du *et al.*, 2016 ▸).

One approach to addressing this issue is to transfer the silicene sheet to a more useful substrate (Peplow, 2015 ▸). Although successful cases have been demonstrated (Martella *et al.*, 2020 ▸), this process is not trivial. A more straightforward approach is to grow silicene on a non-metallic substrate that favors the 2D growth of Si in a hexagonal (honeycomb) arrangement. In this regard, molybdenum disulfide (MoS_2_) emerges as a strong candidate (Fan *et al.*, 2018 ▸) and has already been successfully used to grow silicene (Chiappe *et al.*, 2014 ▸). However, substrate-induced shrinkage of the silicene unit cell has been observed, which is problematic, since silicene’s electronic band structure is sensitive to structural deformation, becoming metallic when the strain reaches 7.5% (Fan *et al.*, 2018 ▸). Also, it is unexpected that lattice mismatch between van der Waals-bonded layered materials would affect their unit-cell dimensions (Shi *et al.*, 2012 ▸), raising questions about the nature of the interaction between MoS_2_ and silicene.

Here, we discuss the growth of silicene on MoS_2_ using molecular dynamics (MD) simulations. The resulting structural characteristics are analyzed and compared with existing data, underscoring the challenges of assessing silicene properties both computationally and experimentally. Silicene stability is achieved only when forces beyond van der Waals interactions are considered, even when modeling a heterostructure of Si atoms intercalated between MoS_2_ layers.

## 2. Methodology

Molecular dynamics simulations were performed using the open-source code *Large-scale Atomic/Molecular Massively Parallel Simulator* (*LAMMPS*) (Thompson *et al.*, 2022 ▸). It is worth noting that for *LAMMPS* to simulate the temporal evolution of a system, it relies on external interatomic potentials. These potentials define the interactions between atoms and molecules, forming the basis for calculating the forces and energies that govern the motion of particles in the system. Unfortunately, there is a scarcity of available potentials in the literature, which restricts the study of many systems. To overcome this limitation, a common approach is the use of hybrid potentials (Barboza *et al.*, 2024*a* ▸), which is the method used in this work. The pair interactions between Mo—Si and S—Si were modeled using a Lennard–Jones (LJ) potential in the following form (Maghfiroh *et al.*, 2020 ▸),
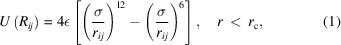
where *U*(*r*_*ij*_) is the potential energy between two atoms *i* and *j*, *r*_*ij*_ is the distance between atoms *i* and *j*, *r*_c_ is the cut radius, σ is a distance parameter and ε is a parameter expressing the strength of the interaction. Both σ and ε are calculated using the Lorentz–Berthelot combining rules, 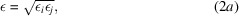

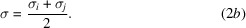
The values for ε_*i*_, ε_*j*_, σ_*i*_, σ_*j*_ and *r*_c_ were chosen as described in the OpenKim repository (Elliott & Lennard-Jones, 2018 ▸; Elliott & Tadmor, 2011 ▸).

The interaction between Si atoms was modeled by an angular dependent potential (ADP). For the ADP, the potential energy is expressed as (Mishin *et al.*, 2005 ▸)
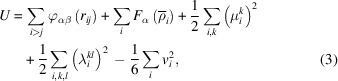
where 
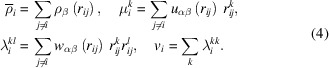
The superscripts *k*, *l* = 1, 2, 3 refer to the Cartesian components. The indices α and β are the element types of the atoms. The first term in equation (3) represents interactions between atoms with a pair potential φ. *F* is the embedding energy that is a function of the total electron density 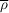. μ and λ represent distortions of the local atomic environment and *v*_*i*_ are traces of the λ tensor. All ADP parameters used in this work were parameterized by Starikov *et al.* (2020 ▸).

Finally, the interactions between Mo—S, Mo—Mo and S—S are dictated by a reactive force-field potential (ReaxFF), where the energy contributions are summarized by the following (Senftle *et al.*, 2016 ▸),
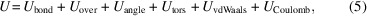
where *U*_bond_ describes the energy associated with forming bonds between atoms, *U*_over_ is an energy penalty preventing the over-coordination of atoms, *U*_angle_ and *U*_tors_ are the energies associated with three-body valence-angle strain and four-body torsion-angle strain, respectively, and *U*_vdWaals_ and *U*_Coulomb_ are dispersive and electrostatic contributions calculated between all atoms, respectively. Full functional forms of ReaxFF can be found elsewhere (Senftle *et al.*, 2016 ▸). Each of these energy components for MoS_2_ was parameterized by Ostadhossein *et al.* (2017 ▸).

**Figure 1:**
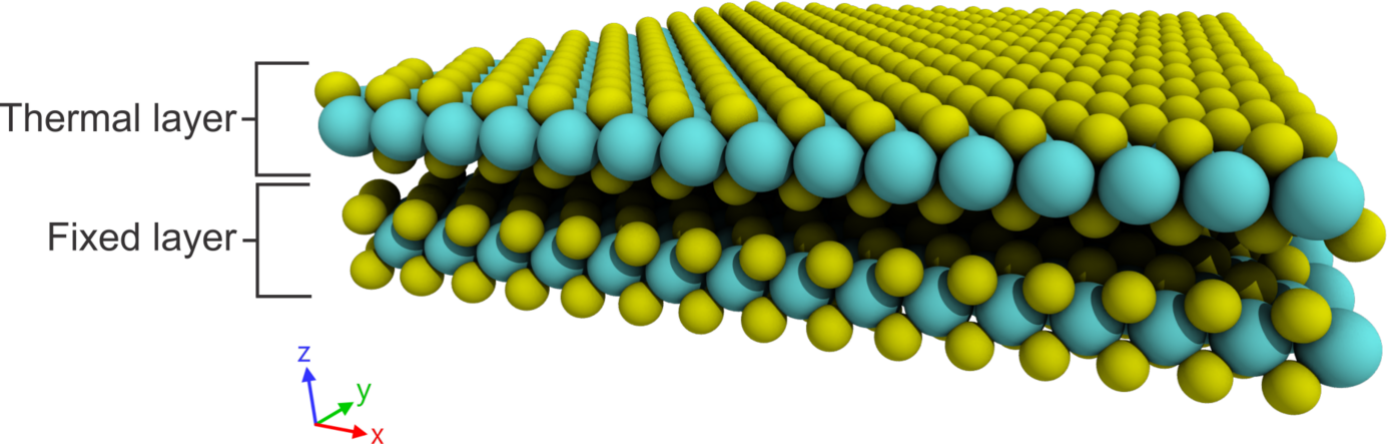
MoS_2_ substrate configuration. Cyan and green spheres represent Mo and S atoms, respectively.

The MoS_2_ substrate was created with the 2*H*-phase, as this is found naturally and is the most commonly observed structure (also known as the *AA*′ phase in the bilayer case) (Yan *et al.*, 2017 ▸), using the open-source software *Atomsk* (Hirel, 2015 ▸). The substrate is composed of two MoS_2_ slabs, where the bottom layer is fixed to function as the catalyst, while the top layer, considered the thermal layer, is responsible for maintaining the substrate temperature at the desired value, as shown in Fig. 1 ▸. To prevent Si atoms from exiting the simulation box, a reflecting wall is implemented at the upper boundary of the simulation cell in the *z* direction. A periodic boundary condition with a rectangular supercell of approximately 5.0 × 5.0 nm is applied to mimic the semi-infinite surface along the *x* and *y* directions. As demonstrated in similar studies, this surface area is sufficient to support proper epitaxial growth (Arifin *et al.*, 2016 ▸; Xu *et al.*, 2016 ▸). A vacuum of 1.0 nm is set along the *z* axis, beyond the last S atom, with no periodic boundary condition, to allow Si atoms to be randomly deposited at a fixed rate of 1 atom.ps^−1^. Thermal layer temperatures of 100, 300, 600 and 800 K were maintained by a Nose/Hoover thermostat under an NVT ensemble, while Si atoms were controlled by an NVE ensemble. The newly deposited Si atoms were positioned at least 2 Å away from existing atoms to prevent overlap. All system visualizations were carried out using the *Ovito* software (Stukowski, 2009 ▸).

## 3. Results and discussion

### 3.1. Silicene structural characteristics

**Figure 2:**
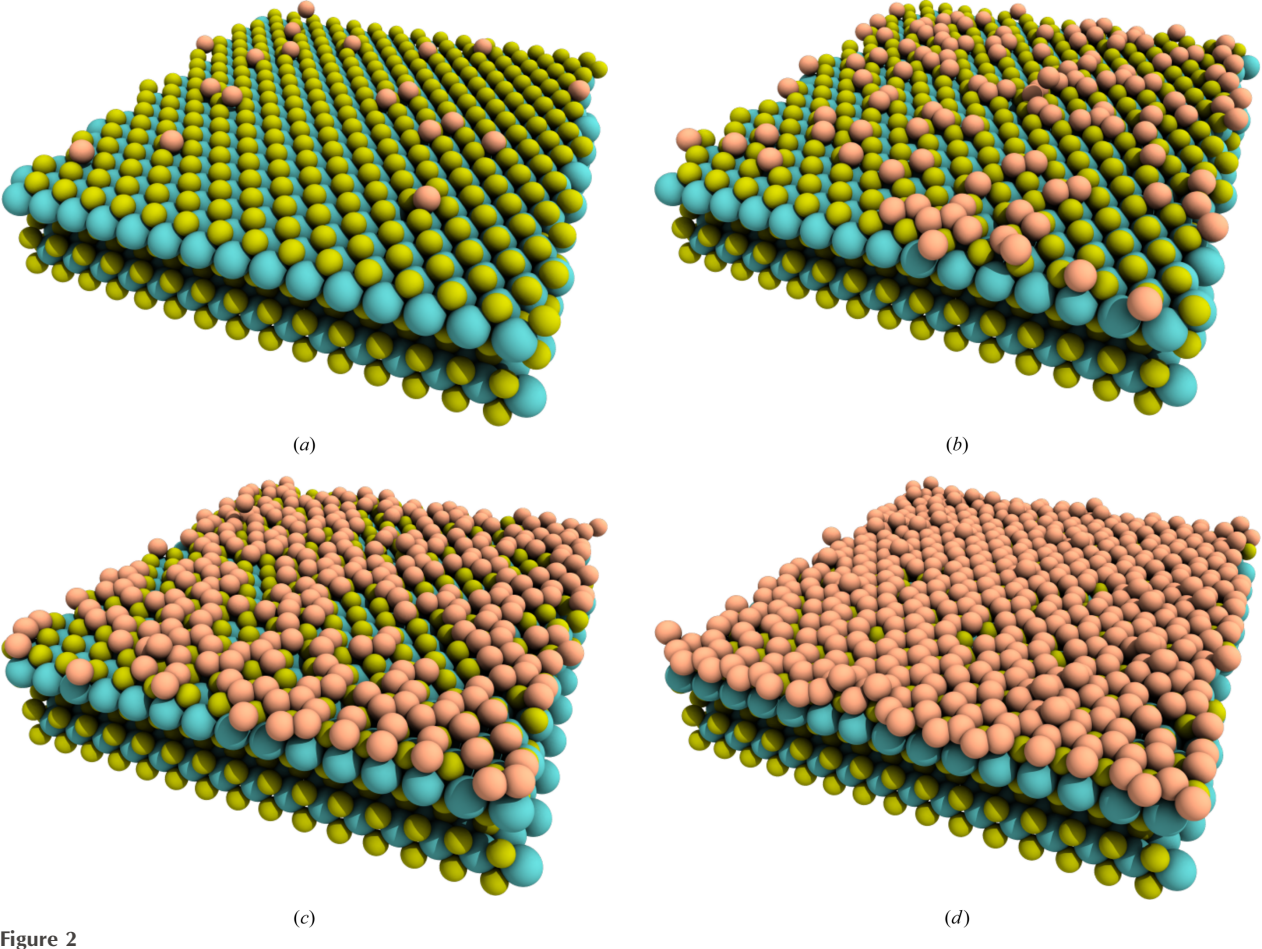
Structural evolution of silicene growth, at 100 K, on MoS_2_ at time intervals of (*a*) 19, (*b*) 117, (*c*) 307 and (*d*) 550 ps. Si atoms are represented as light-orange spheres.

The structural evolution of silicene during epitaxial growth, using the system at 100 K as an example, is shown in Fig. 2 ▸. In the early stages of growth, with a low number of Si atoms, the atoms remain isolated, *i.e.* there is no formation of Si clusters. However, as Si deposition continues, some clusters begin to nucleate, as seen in Fig. 2 ▸(*b*). This tendency persists until the concentration of Si atoms on the substrate surface becomes too high for further nucleation, leading to the coalescence of existing clusters, as depicted in Fig. 2 ▸(*c*). Eventually, the substrate surface becomes nearly fully covered with Si atoms, forming a single silicene layer, as shown in Fig. 2 ▸(*d*). This mechanism is similar to that observed in epitaxial growth on Ir and Au substrates (Barboza *et al.*, 2024*b* ▸; Cherukara *et al.*, 2017 ▸).

**Figure 3:**
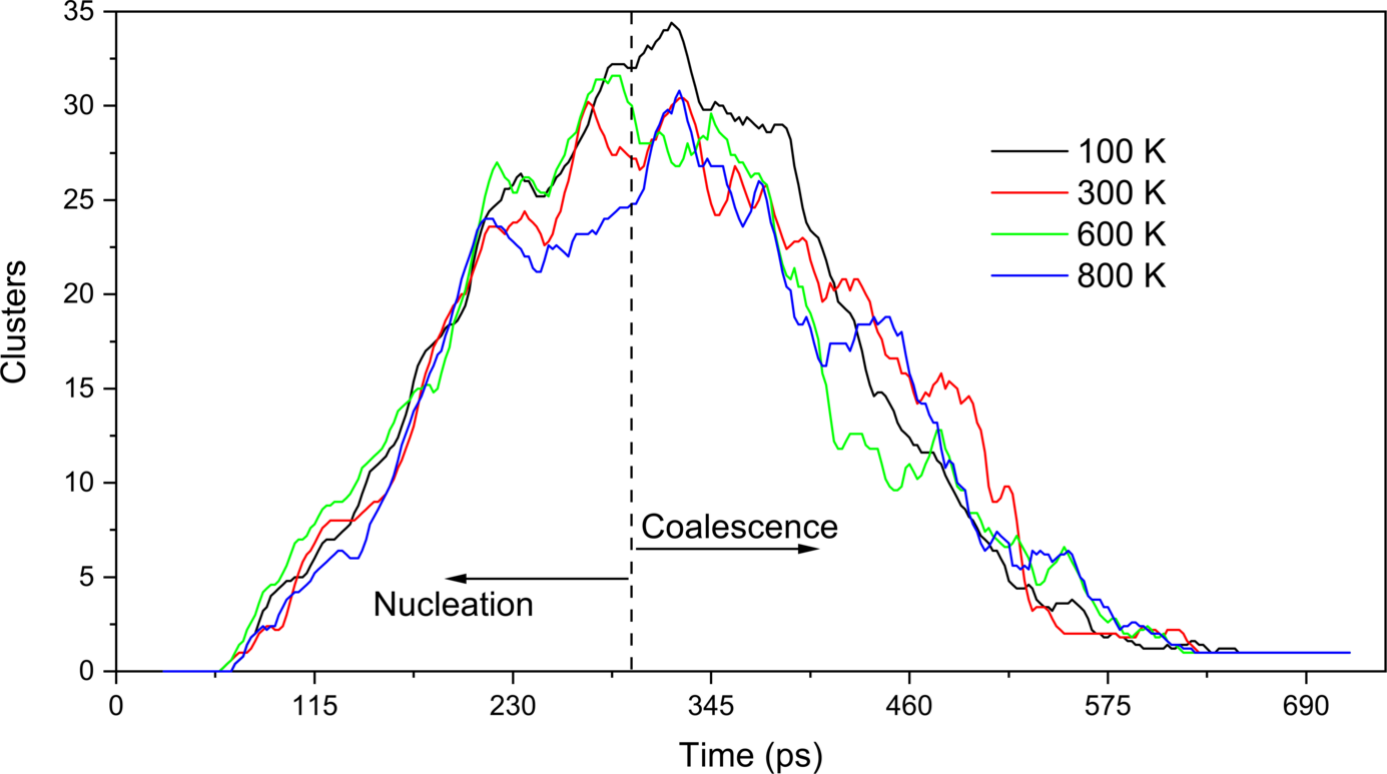
Number of Si clusters as a function of time at 100, 300, 600 and 800 K. The vertical dashed line separates the nucleation and coalescence stages.

In order to quantify and verify whether temperature influences cluster formation, the number of clusters is plotted in Fig. 3 ▸ as a function of time for different temperatures. Although all the curves follow the previously observed pattern of cluster nucleation and coalescence, no clear pattern is evident between them, indicating that the differences observed across all temperatures are merely due to initial stochastic effects introduced by thermal vibrations.

**Figure 4:**
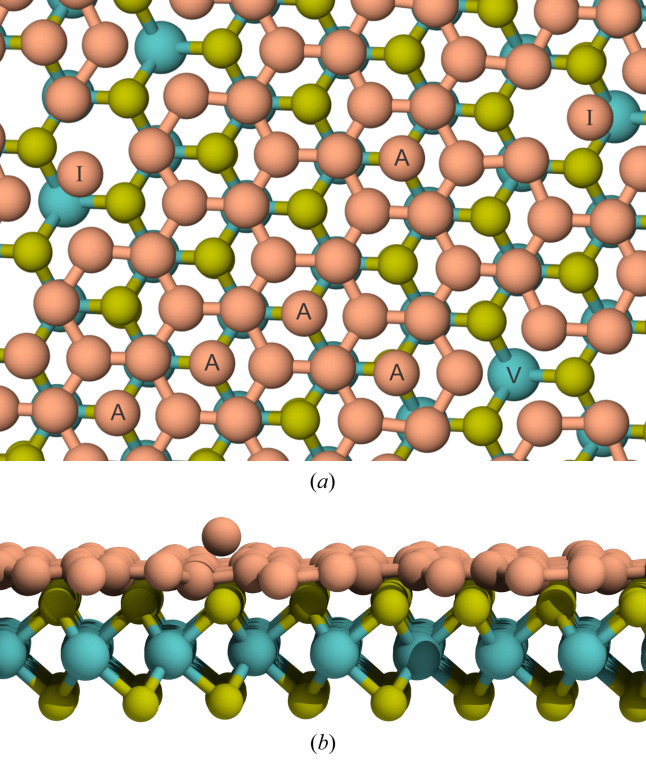
(*a*) Silicene atomic arrangement highlighting the *AB* stacking, perpendicular to the *z* direction, and showing adatom (A), vacancy (V) and isolated (I) Si atom defects. (*b*) A view normal to the *xy* plane. The MoS_2_ thermal layer has been removed in both figures to ease visualization.

As observed in Fig. 2 ▸(*a*), Si atoms tend to adsorb between the valleys formed by S atoms, indicating that the final silicene atomic arrangement on top of MoS_2_ is of Bernal (*AB*) type. It is worth noting that silicene on MoS_2_ can theoretically form three different types of arrangement (*AA*, intermediate and *AB*) with respect to the underlying Mo and S atoms, all of which are equally stable (degenerate) (Houssa *et al.*, 2015 ▸; Scalise *et al.*, 2018 ▸). A better view of the silicene atomic arrangement is presented in Fig. 4 ▸, where it can be seen that S atoms are positioned at the center of Si rings, forming an *AB* structure. Note that this stacking pattern considers only the silicene layer and the MoS_2_ monolayer directly above it. Some defect types are labeled in Fig. 4 ▸, such as vacancies (V), adatoms (adsorbed atoms on a crystal surface) (A) and isolated atoms (I), as these are common occurrences in the experimental synthesis of 2D materials.

The Si adatoms continue diffusing across the silicene structure until they find a stable site, either at available dangling bonds or atop hollow sites in six-membered Si rings. When they encounter dangling bonds, their potential energy decreases depending on the number of atoms already present in the ring. As expected, the potential energy is lower when they find dangling bonds on five-membered rings, as this results in a final ring configuration that is as stable as possible (a six-membered ring). However, if an Si adatom diffuses onto an already complete six-membered ring, it may either stay atop the ring or diffuse to another location. This occurs because the hollow site formed by a six-membered Si ring is metastable. Specifically, the potential energy of an adatom atop a six-membered ring is close to the energy required for the adatom to migrate across the hollow site and reach the opposite side (Barboza *et al.*, 2024*b* ▸). This diffusion energy is calculated to be 1.1 eV, which is relatively high compared with other 2D materials (Jiang *et al.*, 2023 ▸; Kidd *et al.*, 2021 ▸; Shekh *et al.*, 2023 ▸). However, in a dynamic system, such as in this work, this value can be significantly influenced by several factors, such as interactions with other defects, the type of underlying rings, different localized buckling heights and thermal vibrations. Among these factors, thermal vibration is the only one that can be controlled. Since it is known that diffusion rates increase with temperature, one might expect adatom defects, and potentially other defects, to decrease with increasing temperature. To confirm this premise, the total number of defects, including adatoms and non-six-membered rings, was calculated and the results are shown in Table 1 ▸. As expected, there is an overall reduction in defects with increasing temperature.

The interlayer distance, defined as the distance between S atoms and the silicene layer, is 1.1 Å, deviating from the experimental value of 3.0 Å (Chiappe *et al.*, 2014 ▸). It is important to note that the interlayer distance is a very challenging property to simulate, with even first-principles calculations providing discrepant values (Zhu & Schwingenschlögl, 2015 ▸). Similarly, the buckling height is also a complex characteristic to reproduce computationally. Our results show a buckling height of approximately 0.23 Å, indicating a low-buckled lattice. Interestingly, temperature variations do not significantly affect this value. Just as with the interlayer distance, the buckling height value differs from density functional theory (DFT) studies, which report values ranging from 0.45 to 0.54 Å (Kharadi *et al.*, 2021 ▸; Zhu & Schwingenschlögl, 2015 ▸). An important consideration is that the previously mentioned DFT studies model a pristine silicene sheet; that is, the substrate and silicene are created separately and then brought together for energy minimization before starting the simulation. In contrast, our approach involves growing the silicene atom by atom, resulting in a sheet with numerous defects, which is more representative of experimental synthesis. These differences in simulation setup may help explain the variations in the results.

### 3.2. Interactions between Si/MoS_2_ layers

An interesting observation is that, since the stacking pattern found is *AB*, the Si layer must replicate the lattice constant of the MoS_2_ surface. This implies a lattice mismatch between MoS_2_ (lattice constant of 3.16 Å; Reshak & Auluck, 2003 ▸) and free-standing silicene (lattice constant of 3.8 Å; Zólyomi *et al.*, 2018 ▸). The described effect has also been observed experimentally (Chiappe *et al.*, 2014 ▸).

To explain this observation, there must be a strong interaction between MoS_2_ and Si, which is unexpected, as MoS_2_ is typically considered a van der Waals material. However, there is evidence of interlayer charge transfer between silicene and MoS_2_ (Li *et al.*, 2014 ▸), suggesting that interactions beyond van der Waals forces are involved between the stacked layers.

The LJ potential parameters used in this work were obtained from the OpemKIM repository (Elliott & Lennard-Jones, 2018 ▸; Elliott & Tadmor, 2011 ▸). They implicitly consider stronger bonds between Si—Mo and Si—S than just van der Waals forces. To investigate this subject further, we performed additional simulations using parameters from the well known universal force field (UFF) (Rappe *et al.*, 1992 ▸). The UFF includes various types of interaction, such as two-, three- and four-body interactions, as well as bonded and non-bonded interactions. Since our focus is on non-bonded interactions, all bonded contributions were disregarded, effectively considering only van der Waals interactions modeled using the LJ approach. As usual, the Lorentz-Berthelot mixing rules [equations (2*a*) and 2(*b*)] were applied to estimate the parameters between non-identical atom pairs.

With this new set of parameters, the Si atoms self-assembled into a 3D structure, resembling the configuration of free-standing silicene after thermal equilibrium (van Bremen *et al.*, 2017 ▸). Therefore, van der Waals forces alone indeed seem insufficient to maintain the integrity of silicene, suggesting the presence of additional interlayer forces between the silicene and MoS_2_ layers. This finding aligns with studies using MoTe_2_ as substrate (Szary, 2019 ▸), where, despite interactions typically being dominated by van der Waals forces, the silicene/MoTe_2_ interface forms a well defined and energetically favorable configuration when covalent bonds are present. Conversely, unfavorable configurations arise when interactions are weak and solely governed by van der Waals forces.

**Figure 5:**
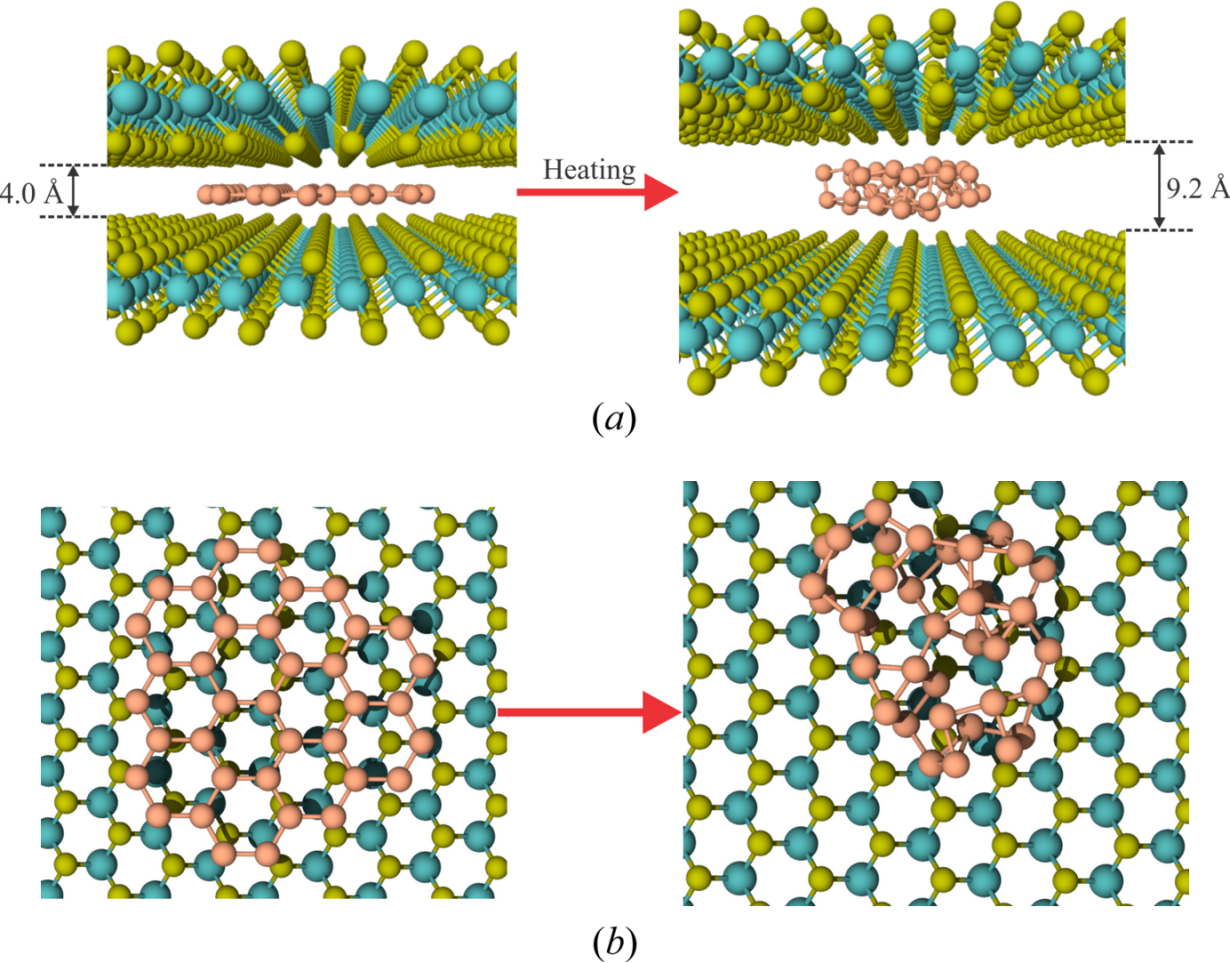
Configuration of the setup where silicene is intercalated between two MoS_2_ layers, with (*a*) a side view, normal to the *xy* plane, and (*b*) a top view. The red arrows indicate the corresponding configurations after heating.

To gain a deeper insight regarding the interaction forces between silicene and MoS_2_, a new configuration was modeled based on the findings of van Bremen *et al.* (2017 ▸). In this setup, the deposited Si atoms do not reside directly on the MoS_2_ surface but rather intercalate between the MoS_2_ layers. This implies that Si atoms must diffuse through the substrate via cracks, wrinkles or edges. Such diffusion does not occur in our models, as the substrate is assumed to be pristine (without cracks or wrinkles) and to have periodic boundaries (no edges). Nevertheless, it is reasonable to assume that a few Si atoms may have diffused through the substrate, forming an initial silicene cluster, as shown in Fig. 5 ▸(*a*). Two modifications are necessary to adapt this configuration for our purposes. First, the previously fixed MoS_2_ layer is now treated as a thermal layer, as it will interact with the silicene. Second, the interlayer spacing between the MoS_2_ layers is increased to accommodate the silicene.

In this scenario, during the heating process the silicene sheet causes the MoS_2_ layers to expand, separating them by 9.2 Å (Fig. 5 ▸) and forming a 3D structure similar to what is observed in epitaxial growth driven solely by van der Waals forces. Consequently, we conclude that the deposited Si atoms do not intercalate between the MoS_2_ layers but instead remain on the MoS_2_ surface, consistent with the experimental findings of Chiappe *et al.* (2014 ▸). However, this conclusion contradicts the evidence reported by van Bremen *et al.* (2017 ▸), where a stable silicene configuration with an interlayer MoS_2_ distance of 6.52 Å was observed.

These inconsistencies across various studies underscore the complexity of silicene growth on MoS_2_. On the one hand, experimental work is hindered by the inherent challenges of studying materials on such a small scale. Techniques like scanning tunneling microscopy or atomic force microscopy may lack the resolution or sensitivity required to detect certain interfacial phenomena or subtle structural changes, leading to differing interpretations. On the other hand, theoretical studies are limited by the inability of computational models to capture fully the range of physical, chemical and electronic properties necessary to predict accurately and characterize unambiguously the silicene on MoS_2_. Moreover, factors like defects, strain, interatomic potential or substrate interactions may not be adequately considered, contributing to variations in results from different computational approaches.

More specifically, van der Waals forces are cumulative over large lateral extents; in classical MD these contributions depend on the interaction cutoff, as well as on lateral size and substrate thickness. In this work we did not perform a full lateral-size/thickness convergence study and therefore we cannot strictly exclude finite-size effects in the adhesion energetics. To mitigate this partially, we tested two distinct parameter sets, OpenKIM (Elliott & Lennard-Jones, 2018 ▸) and UFF (Rappe *et al.*, 1992 ▸), and observed that purely van der Waals interactions drive silicene into a 3D disordered state, whereas potentials with stronger Si—Mo/Si—S inter­actions stabilize a 2D low-buckled silicene. For quantitative interaction energies, however, further convergence tests (including MD with larger lateral size and DFT+vdW correction) are required and constitute an important direction for future work.

## 4. Conclusions

The structural characteristics of silicene grown on an MoS_2_ substrate have been studied using molecular dynamics simulations. The growth mechanism, involving the formation and coalescence of Si clusters, resembles that observed on other substrates like Au and Ir. This process includes defect formation, which can be significantly minimized by increasing the substrate temperature. The stable silicene structure exhibits a low-buckled lattice, with a buckling height of approximately 0.23 Å and an interlayer distance of 1.1 Å between S and Si atoms.

The resulting stacking pattern is of the *AB* type, indicating a lattice mismatch between MoS_2_ and silicene. Such an arrangement cannot be explained solely by van der Waals forces. The LJ potential initially employed to model Si—Mo and Si—S pairs also incorporates bonded interactions. When these bonded interactions are excluded, the silicene structure becomes unstable, indicating that van der Waals forces alone are insufficient to preserve its integrity. Even considering a heterostructure where silicene is sandwiched between two MoS_2_ sheets, potentially improving the mechanical stability, the silicene still becomes unstable, collapsing into a 3D disarray of Si atoms and causing expansion of the MoS_2_.

The complex nature of silicene grown on MoS_2_ presents a significant challenge in evaluating its properties and understanding the underlying physical phenomena using both experimental and computational approaches. This has led to a wide range of results in the literature, many of which often contradict one another. Although our work represents one of the first, if not the very first, large-scale studies of silicene characteristics on MoS_2_ grown via a vapor-deposition-like method, additional analyses are still necessary. In particular, the development of a more accurate interatomic potential would represent a significant step forward. However, it is worth noting that even DFT results remain inconsistent in this subject, hindering progress in this context.

## Figures and Tables

**Table 1:** Number of defects as a function of temperature and ring type

	Temperature (K)			
Defect type	100	300	600	800
Adatom	42	31	28	21
Three-membered ring	206	142	126	86
Four-membered ring	0	6	6	9
Five-membered ring	6	22	25	39
Seven-membered ring	0	2	1	2
Eight-membered ring	37	10	12	3
Total number of defects	291	213	198	160

## Data Availability

The data supporting the findings of this study have been deposited with Zenodo (https://zenodo.org/records/17560338).
